# Supplementary material for: Characteristics and clinical outcomes of patients with kidney failure of unknown aetiology from ANZDATA registry
Source: PLoS One. 2024 Mar 11;19(3):e0300259. doi: 10.1371/journal.pone.0300259 (PMC10927112; doi:10.1371/journal.pone.0300259)
Supplement: S1 Table — (DOCX) [file pone.0300259.s001.docx]

**Table S1: Frequencies of primary kidney diseases making up “other” group in dialysis and transplant cohorts**

| **Cause of kidney failure** | **Dialysis group** | **Transplant group** |
| --- | --- | --- |
| Other (specify) | 1543 | 468 |
| Lead nephropathy | 66 | 16 |
| Cadmium Toxicity | 5 | 2 |
| Renal tuberculosis | 42 | 8 |
| Amyloid disease | 581 | 38 |
| Cortical necrosis | 190 | 93 |
| Interstitial nephritis | 670 | 280 |
| Congenital renal hypoplasia and dysplasia | 115 | 344 |
| Loss of single kidney (trauma-surgery) | 98 | 34 |
| Megaureter | 9 | 7 |
| Oxalosis | 42 | 11 |
| Cystinosis | 5 | 34 |
| Balkan Nephropathy | 6 | 4 |
| Renal Cell Carcinoma (GRAWITZ) | 398 | 52 |
| Transitional Cell Carcinoma Urinary Tract | 150 | 12 |
| Paraproteinaemia (Including Multiple Myeloma) | 1034 | 9 |
| Light Chain Nephropathy (Not Malignant) | 77 | 13 |
| Lithium Toxicity | 346 | 91 |
| Post Partum Nephropathy | 10 | 16 |
| Sarcoidosis | 26 | 14 |
| Posterior Urethral Valves | 15 | 150 |
| Pelvi-Ureteric Junction Obstruction | 34 | 23 |
| Obstructed Megaureter | 12 | 6 |
| Neuropathic Bladder | 52 | 49 |
| Non-obstructed dilated bladder (megacystitis-megaureter) |  | 6 |
| Spina Bifida or Myelomeningocoele | 37 | 47 |
| Bladder Neck Obstruction (incl. Prostatomegaly) | 127 | 16 |
| Other Lower Urinary Tract Abnormalities (with Secondary Reflux) | 43 | 64 |
| Ureteric Obstructive Nephropathy | 200 | 38 |
| Obstructive Nephropathy | 542 | 98 |
| Calcineurin Inhibitor Toxicity | 188 | 50 |
| Scleroderma | 188 | 26 |
| Analgesic Nephropathy | 1840 | 217 |
| Renal Vascular Disease (Malignant Hypertension) | 666 | 206 |
| Renal Vascular Disease-Type Unspecified | 2269 | 141 |
| Renal Vascular Disease-Hypertension (Nephrosclerosis) | 5247 | 807 |
| Atheroembolic Disease (Cholesterol Emboli) | 331 | 6 |
| Bilateral Renal Artery Stenosis | 495 | 26 |
| Medullary Cystic Disease | 50 | 171 |
| Infantile/Juvenile Polycystic Kidney Disease | 15 | 45 |
| Reflux Nephropathy | 878 | 1701 |
| Pyelonephritis | 63 | 15 |
| Calculi | 405 | 83 |
| Gout | 69 | 24 |
| Missing | 478 | 172 |
