# Supplementary material for: Characteristics and clinical outcomes of patients with kidney failure of unknown aetiology from ANZDATA registry
Source: PLoS One. 2024 Mar 11;19(3):e0300259. doi: 10.1371/journal.pone.0300259 (PMC10927112; doi:10.1371/journal.pone.0300259)
Supplement: S2 Table — (DOCX) [file pone.0300259.s002.docx]

**Table S2: Characteristics and medical conditions of the dialysis cohort**

| **Characteristics** | **uESKD**  **N= 3640** | **DN**  **N=24,513** | **GN**  **N=10,450** | **ADPKD**  **N=2188** | **Other**  **N= 19,657** | **P-value** |
| --- | --- | --- | --- | --- | --- | --- |
| **Age (years)** |  |  |  |  |  | <0.001* |
| <20 | 17 (0.5) | 4 (0.0) | 116 (1.1) | 7 (0.3) | 139 (0.7) |  |
| 20-39 | 190 (5.2) | 1734 (7.1) | 1190 (11.4) | 80 (3.7) | 863 (4.4) |  |
| 40-59 | 731 (20.1) | 9195 (37.5) | 2924 (28.0) | 732 (33.5) | 3575 (18.2) |  |
| 60-79 | 2171 (59.6) | 12546(51.2) | 5542(53.0) | 1262 (57.7) | 12441 (63.3) |  |
| 80+ | 531 (14.6) | 1034 (4.2) | 678 (6.5) | 107 (4.9) | 2639 (13.4) |  |
| **Gender** |  |  |  |  |  | <0.001* |
| Female | 1430 (39.3) | 10057 (41.0) | 3844 (36.8) | 997 (45.6) | 8110 (41.3) |  |
| Male | 2210 (60.7) | 14456 (59.0) | 6606 (63.2) | 1191 (54.4) | 11547 (58.7) |  |
| **Smoking status** |  |  |  |  |  | <0.001* |
| Never | 1627 (44.7) | 10567 (43.1) | 4426 (42.4) | 1064 (48.6) | 8429 (42.9) |  |
| Former | 1438 (39.5) | 10026 (40.9) | 4102 (39.3) | 804 (36.7) | 8138 (41.4) |  |
| Current | 478 (13.1) | 3376 (13.8) | 1580 (15.1) | 252 (11.5) | 2342 (11.9) |  |
| Missing | 97 (2.7) | 544 (2.2) | 342 (3.3) | 68 (3.1) | 748 (3.8) |  |
| **BMI** (kg/m^2^) |  |  |  |  |  | <0.001* |
| <18.5 | 194 (5.3) | 286 (1.2) | 424 (4.1) | 68 (3.1) | 930 (4.7) |  |
| 18.5-24.9 | 1289 (35.4) | 5306 (21.6) | 3653 (35.0) | 839 (38.3) | 7250 (36.9) |  |
| 25-29.9 | 1083 (29.8) | 7084 (28.9) | 3012 (28.8) | 662 (30.3) | 5792 (29.5) |  |
| >30 | 850 (23.4) | 10759 (43.9) | 2786 (26.7) | 506 (23.1) | 4423 (22.5) |  |
| Missing | 224 (6.2) | 1078 (4.4) | 575 (5.5) | 113 (5.2) | 1262 (6.4) |  |
| **Ethnicity** |  |  |  |  |  | <0.001* |
| White | 2689 (73.9) | 12185 (49.7) | 7483 (71.6) | 1909 (87.2) | 16104 (81.9) |  |
| ATSI | 343 (9.4) | 3874 (15.8) | 740 (7.1) | 22 (1.0) | 759 (3.9) |  |
| Māori | 237 (6.5) | 5409 (22.1) | 948 (9.1) | 72 (3.3) | 1025 (5.2) |  |
| Asian | 253 (7.0) | 2332 (9.5) | 1014 (9.7) | 118 (5.4) | 1093 (5.6) |  |
| Other | 57 (1.6) | 503 (2.1) | 186 (1.8) | 49 (2.2) | 357 (1.8) |  |
| Missing | 61 (1.7) | 210 (0.9) | 79 (0.8) | 18 (0.8) | 319 (1.6) |  |
| **Comorbidities** |  |  |  |  |  |  |
| Peripheral vascular disease | 713 (19.6) | 9557 (39.0) | 1599 (15.3) | 250 (11.4) | 5009 (25.5) | <0.001* |
| Cerebrovascular disease | 563 (15.5) | 4170 (17.0) | 1224 (11.7) | 272 (12.4) | 3469 (17.6) | <0.001* |
| Coronary artery disease | 1528 (42.0) | 12378 (50.5) | 3509 (33.6) | 653 (29.8) | 8856 (45.1) | <0.001* |
| Chronic lung disease | 729 (20.0) | 4181 (17.1) | 2030 (19.4) | 281 (12.8) | 4065 (20.7) | <0.001* |
| Diabetes mellitus | 883 (24.3) | 24504 (100.0) | 1916 (18.3) | 246 (11.2) | 4094 (20.8) | <0.001* |
| **Dialysis modality** |  |  |  |  |  | <0.001* |
| Haemodialysis | 2638 (72.5) | 17610 (72.3) | 7427 (71.1) | 1461 (67.3) | 14481 (73.8) |  |
| Peritoneal dialysis | 999 (27.5) | 6741 (27.7) | 3012 (28.9) | 710 (32.7) | 5149 (26.2) |  |
| **Dialysis vintage** |  |  |  |  |  | <0.001* |
| 1972-1998 | 675 (18.5) | 2680 (10.9) | 2300 (22.0) | 464 (21.2) | 3449 (17.5) |  |
| 1999-2008 | 1289 (35.4) | 7285 (29.7) | 3437 (32.9) | 603 (27.6) | 6173 (31.4) |  |
| 2009-2018 | 1239 (34.0) | 11463 (46.8) | 3568 (34.1) | 815(37.2) | 7735 (39.3) |  |
| 2018-2021 | 437 (12.0) | 3085 (12.6) | 1145 (11.0) | 306 (14.0) | 2300 (11.7) |  |
| **Mortality rate** |  |  |  |  |  |  |
| 1 year mortality | 866 (31.6) | 4317 (25.6) | 1600 (21.8) | 236 (17.2) | 4614 (31.8) | <0.001* |
| 3 year mortality | 1609 (58.7) | 9536 (56.6) | 3482 (47.4) | 555 (40.5) | 8838 (60.9) | <0.001* |
| 5 year mortality | 2116 (77.2) | 12880 (76.4) | 4909 (66.8) | 827 (60.4) | 11369 (78.4) | <0.001* |
| **Cause of mortality^a^** |  |  |  |  |  | <0.001* |
| Cardiovascular | 1015 (37.1) | 7807 (46.7) | 2817 (38.5) | 561 (41.1) | 5153 (35.7) |  |
| Infection | 322 (11.8) | 1941 (11.6) | 879 (12.0) | 149 (10.9) | 1493 (10.3) |  |
| Withdrawal | 787 (28.8) | 3896 (23.3) | 1877 (25.7) | 381 (27.9) | 4311 (29.9) |  |
| Cancer | 126 (4.6) | 432 (2.6) | 370 (5.1) | 70 (5.1) | 1059 (7.3) |  |
| Other | 484 (17.8) | 2638 (15.8) | 1365 (18.7) | 205 (15.0) | 2413 (16.7) |  |
| **Biopsy proven** |  |  |  |  |  | <0.001* |
| No | 2948 (81.0) | 19733 (80.5) | 2919 (27.9) | 1928 (88.1) | 13154 (66.9) |  |
| Yes | 260 (7.1) | 2925 (11.9) | 7010 (67.1) | 110 (5.0) | 4102 (20.9) |  |
| Missing | 432 (11.9) | 1855 (7.6) | 521 (5.0) | 150 (6.9) | 2401 (12.2) |  |
| **Abbreviations**: ADPKD = autosomal dominant polycystic kidney disease, ATSI = Aboriginal and Torres Strait Islander, BMI = body mass index, DN = diabetic nephropathy, GN = glomerular disease, uESKD = kidney failure of unknown aetiology  χ2 tests of independence reported with Bonferroni correction for multiple testing; *p<0.005 considered statistically significant  ^a^ percentages calculated as proportion of people who died during follow-up | | | | | | |
