# Supplementary material for: Characteristics and clinical outcomes of patients with kidney failure of unknown aetiology from ANZDATA registry
Source: PLoS One. 2024 Mar 11;19(3):e0300259. doi: 10.1371/journal.pone.0300259 (PMC10927112; doi:10.1371/journal.pone.0300259)
Supplement: S3 Table — (DOCX) [file pone.0300259.s003.docx]

**Table S3:** **Characteristics and medical conditions of the transplant cohort**

| **Characteristics** | **uESKD**  **N=880** | **DN**  **N=2458** | **GN**  **N=8957** | **ADPKD**  **N=2831** | **Other**  **N= 5733** | **P-value** |
| --- | --- | --- | --- | --- | --- | --- |
| **Age (years)** |  |  |  |  |  | <0.001* |
| <20 | 45 (5.1) | 0 (0) | 583 (6.5) | 14 (0.5) | 1002 (17.5) |  |
| 20-39 | 256 (29.1) | 349 (14.2) | 3153 (35.2) | 250 (8.8) | 1657 (28.9) |  |
| 40-59 | 397 (45.1) | 1523 (62.0) | 4093 (45.7) | 2034 (71.8) | 2165 (37.8) |  |
| 60-79 | 182 (20.7) | 586 (23.8) | 1128 (12.6) | 533 (18.8) | 908 (15.8) |  |
| 80+ | 0 (0) | 0 (0) | 0 (0) | 0 (0) | 1 (0.0) |  |
| **Gender** |  |  |  |  |  | <0.001* |
| Female | 317 (36.0) | 716 (29.1) | 3093 (34.5) | 1244 (43.9) | 2410 (42.0) |  |
| Male | 563 (64.0) | 1742 (70.9) | 5864 (65.5) | 1587 (56.1) | 3323 (58.0) |  |
| **Smoking status** |  |  |  |  |  | <0.001* |
| Never | 485 (55.1) | 1171 (47.6) | 5052 (56.4) | 1630 (57.6) | 3470 (60.5) |  |
| Former | 261 (29.7) | 962 (39.1) | 2702 (30.2) | 923 (32.6) | 1448 (25.3) |  |
| Current | 98 (11.1) | 246 (1.0) | 903 (10.1) | 183 (6.5) | 526 (9.2) |  |
| Missing | 36 (4.1) | 79 (3.2) | 300 (3.3) | 95 (3.4) | 289 (5.0) |  |
| **BMI (**kg/m^2^) |  |  |  |  |  | <0.001* |
| <18.5 | 54 (6.1) | 24 (1.0) | 458 (5.1) | 56 (2.0) | 756 (13.2) |  |
| 18.5-24.9 | 323 (36.7) | 572 (23.3) | 3622 (40.4) | 1070 (37.8) | 2133 (37.2) |  |
| 25-29.9 | 263 (29.9) | 819 (33.3) | 2605 (29.1) | 991 (35.0) | 1455 (25.4) |  |
| >30 | 167 (19.0) | 900 (36.6) | 1687 (18.8) | 534 (18.9) | 951 (16.6) |  |
| Missing | 73 (8.3) | 143 (5.8) | 585 (6.5) | 180 (6.4) | 438 (7.6) |  |
| **Ethnicity** |  |  |  |  |  | <0.001* |
| White | 551 (62.6) | 1217 (49.5) | 6446 (72.0) | 2469 (87.2) | 4427 (77.2) |  |
| ATSI | 45 (5.1) | 278 (11.3) | 250 (2.8) | 12 (0.4) | 137 (2.4) |  |
| Māori | 91 (10.3) | 420 (17.1) | 510 (5.7) | 46 (1.6) | 255 (4.4) |  |
| Asian | 122 (13.9) | 383 (15.6) | 1157 (12.9) | 130 (4.6) | 425 (7.4) |  |
| Other | 29 (3.3) | 65 (2.6) | 160 (1.8) | 47 (1.7) | 151 (2.6) |  |
| Missing | 42 (4.8) | 95 (3.9) | 434 (4.8) | 127 (4.5) | 338 (5.9) |  |
| **Comorbidities** |  |  |  |  |  |  |
| Peripheral vascular disease | 24 (2.7) | 584 (23.8) | 233 (2.6) | 69 (2.4) | 211 (3.7) | <0.001* |
| Cerebrovascular disease | 22 (2.5) | 198 (8.1) | 206 (2.3) | 132 (4.7) | 144 (2.5) | <0.001* |
| Coronary artery disease | 74 (8.4) | 684 (27.8) | 689 (7.7) | 247 (8.7) | 490 (8.5) | <0.001* |
| Chronic lung disease | 48 (5.5) | 161 (6.6) | 457 (5.1) | 102 (3.6) | 296 (5.2) | <0.001* |
| Diabetes mellitus | 69 (7.8) | 2454 (99.8) | 433 (4.8) | 98 (3.5) | 309 (5.4) | <0.001* |
| **First KRT modality** |  |  |  |  |  | <0.001* |
| Haemodialysis | 518 (58.9) | 1527 (62.1) | 5562 (62.1) | 1697 (59.9) | 3013 (52.6) |  |
| Peritoneal dialysis | 280 (31.8) | 797 (32.4) | 2536 (28.3) | 724 (25.6) | 1810 (31.6) |  |
| Pre-emptive transplant | 82 (9.3) | 134 (5.5) | 859 (9.6) | 410 (14.5) | 910 (15.9) |  |
| **Dialysis vintage (years, mean, SD)** | 30.3±32.0 | 34.5±27.1 | 30.8±30.6 | 26.7±27.6 | 27.0±29.5 | <0.001*^ |
| **HLA mismatch (mean, SD)** | 3.3±1.63 | 3.6±1.6 | 3.2±1.6 | 3.3± 1.63 | 3.2±1.6 | <0.001*^ |
| **Max PRA (%, mean, SD)** | 8.4±19.9 | 9.0±20.7 | 10.6±21.9 | 10.3±22.0 | 10.0±21.5 | <0.001*^ |
| **Transplant era** |  |  |  |  |  | <0.001* |
| 1989-1998 | 147 (16.7) | 271 (11.0) | 1870 (20.9) | 414 (14.6) | 1134 (19.8) |  |
| 1999-2008 | 233 (26.5) | 495 (20.1) | 2883 (32.2) | 803 (28.4) | 1521 (26.5) |  |
| 2009-2018 | 410 (46.6) | 1277 (52.0) | 3501 (39.1) | 1353 (47.8) | 2509 (43.8) |  |
| 2018-2021 | 89 (10.1) | 415 (16.9) | 703 (7.8) | 261 (9.2) | 569 (9.9) |  |
| **Mortality rate** |  |  |  |  |  |  |
| 1 year mortality | 7 (2.8) | 21 (2.2) | 32 (1.4) | 16 (2.1) | 45 (3.3) | 0.006 |
| 3 year mortality | 34 (13.8) | 106 (11.3) | 161 (7.2) | 61 (7.9) | 138 (10.2) | <0.001* |
| 5 year mortality | 59 (24.0) | 230 (24.6) | 339 (15.2) | 113 (14.6) | 241 (17.8) | <0.001* |
| **Cause of mortality^a^** |  |  |  |  |  | <0.001* |
| Cardiovascular | 71 (29.1) | 397 (42.9) | 674 (30.4) | 194 (25.4) | 405 (30.0) |  |
| Infection | 55 (22.5) | 172 (18.6) | 346 (15.6) | 117 (15.2) | 213 (15.8) |  |
| Withdrawal | 20 (8.2) | 83 (9.0) | 192 (8.7) | 69 (9.0) | 158 (11.7) |  |
| Cancer | 54 (22.1) | 95 (10.3) | 515 (23.2) | 224 (29.3) | 270 (20.0) |  |
| Other | 44 (18.0) | 179 (19.3) | 491 (22.1) | 161 (21.0) | 304 (22.5) |  |
| **Biopsy proven** |  |  |  |  |  | <0.001* |
| No | 641 (72.8) | 1692 (68.8) | 1805 (20.2) | 2653 (93.7) | 3540 (61.7) |  |
| Yes | 152 (17.3) | 489 (19.9) | 7061 (78.8) | 131 (4.6) | 1390 (24.2) |  |
| Missing | 87 (9.9) | 277 (11.3) | 91 (1.0) | 47 (1.7) | 803 (14.0) |  |
| **Abbreviations**: ADPKD = autosomal dominant polycystic kidney disease, ATSI = Aboriginal and Torres Strait Islander, BMI = body mass index, DN = diabetic nephropathy, GN = glomerular disease, PRA = panel reactive antibodies, SD = standard deviation, uESKD = kidney failure of unknown aetiology  χ2 tests of independence reported with Bonferroni correction for multiple testing; *p<0.005 considered statistically significant  ^a^ percentages calculated as proportion of people who died during follow-up  ^ One way ANOVA followed by post hoc test with Bonferroni correction | | | | | | |
