# Supplementary material for: Characteristics and clinical outcomes of patients with kidney failure of unknown aetiology from ANZDATA registry
Source: PLoS One. 2024 Mar 11;19(3):e0300259. doi: 10.1371/journal.pone.0300259 (PMC10927112; doi:10.1371/journal.pone.0300259)
Supplement: S4 Table — (DOCX) [file pone.0300259.s004.docx]

**Table S4: Subgroup analysis evaluating association between kidney disease status and mortality in dialysis cohort**

| **Effect** | **Unadjusted** | | **Adjusted** | |
| --- | --- | --- | --- | --- |
|  | **HR** | **95% CI** | **HR** | **95% CI** |
| **Disease status** |  | |  | |
| uESKD | 1.00 | 0.96-1.04 | 1.00 | 0.96-1.04 |
| Diabetic nephropathy | 0.85*** | 0.84-0.87 | 0.87*** | 0.84-0.90 |
| Glomerular disease | 0.72*** | 0.70-0.75 | 0.73*** | 0.71-0.75 |
| ADPKD | 0.62*** | 0.58-0.65 | 0.61*** | 0.57-0.64 |
| Other | Ref | | Ref | |
| **Gender** |  | |  | |
| Male | Ref | | Ref | |
| Female | 0.93*** | 0.91-0.95 | 0.96*** | 0.94-0.98 |
| **Ethnicity** |  | |  | |
| White | Ref | | Ref | |
| Non-white | 0.75*** | 0.73-0.76 | 0.75*** | 0.73-0.77 |
| **Smoking status** |  | |  | |
| Never | Ref | | Ref | |
| Former | 1.17*** | 1.14-1.19 | 1.13*** | 1.10-1.15 |
| Current | 1.07*** | 1.04-1.11 | 1.06*** | 1.03-1.10 |
| **BMI** (kg/m^2^) |  | |  | |
| <18.5 | Ref | | Ref | |
| 18.5-24.9 | 0.85*** | 0.81-0.90 | 0.83*** | 0.78-0.87 |
| 25-29.9 | 0.81*** | 0.77-0.85 | 0.79*** | 0.74-0.83 |
| >30 | 0.71*** | 0.67-0.75 | 0.71*** | 0.67-0.75 |
| **Diabetes** |  | |  | |
| Absent | Ref | | Ref | |
| Present | 1.03*** | 1.01-1.05 | 1.2*** | 1.16-1.24 |
| **First dialysis modality** |  | |  | |
| Haemodialysis | Ref | | Ref | |
| Peritoneal dialysis | 1.03** | 1.01-1.05 | 1.01 | 0.98-1.0 |
| **Dialysis vintage** |  | |  | |
| 1989-1998 | Ref | | Ref | |
| 1999-2008 | 0.85*** | 0.83-0.87 | 0.90*** | 0.88-0.93 |
| 2009-2018 | 0.67*** | 0.66-0.69 | 0.72*** | 0.70-0.75 |
| 2018-2021 | 0.33*** | 0.31-0.36 | 0.37*** | 0.34-0.40 |
| **Abbreviations**: ADPKD = autosomal dominant polycystic kidney disease, BMI = body mass index, KRT = kidney replacement therapy, ref = reference, uESKD = kidney failure of unknown aetiology  Significance level: *<0.05, **<0.01, ***<0.001 | | | | |
