# Supplementary material for: Characteristics and clinical outcomes of patients with kidney failure of unknown aetiology from ANZDATA registry
Source: PLoS One. 2024 Mar 11;19(3):e0300259. doi: 10.1371/journal.pone.0300259 (PMC10927112; doi:10.1371/journal.pone.0300259)
Supplement: S5 Table — (DOCX) [file pone.0300259.s005.docx]

**Table S5: Subgroup analysis evaluating association between kidney disease status and mortality in transplant cohort**

| **Effect** | **Unadjusted** | | **Adjusted** | |
| --- | --- | --- | --- | --- |
|  | **HR** | **95% CI** | **HR** | **95% CI** |
| **Recipient disease status** |  | |  | |
| uESKD | 1.32*** | 1.15-1.52 | 1.10 | 0.95-1.28 |
| Diabetic nephropathy | 2.7*** | 2.54-3.01 | 1.27** | 1.09-1.48 |
| Glomerular disease | 0.92* | 0.86-0.99 | 0.86*** | 0.80-0.93 |
| ADPKD | 1.31*** | 1.20-1.43 | 0.90* | 0.81-0.99 |
| Other | Ref | | Ref | |
| **Recipient gender** |  | |  | |
| Male | Ref | | Ref | |
| Female | 0.86*** | 0.82-0.91 | 0.96 | 0.90-1.02 |
| **Recipient ethnicity** |  | |  | |
| White | Ref | | Ref | |
| Non-white | 0.82*** | 0.77-0.87 | 0.84*** | 0.78-0.90 |
| **Recipient age** |  | |  | |
| < 20 Years | Ref | | Ref | |
| 20-39 Years | 1.56*** | 1.31-1.86 | 1.51*** | 1.25-1.83 |
| 40-59 Years | 4.11*** | 3.48-4.84 | 4.10*** | 3.40-4.94 |
| 60-79 Years | 8.1*** | 6.89-9.65 | 9.10*** | 7.52-11.1 |
| **Recipient smoking status** |  | |  | |
| Never | Ref | | Ref | |
| Former | 1.67*** | 1.57-1.77 | 1.22*** | 1.14-1.30 |
| Current | 1.75*** | 1.61-1.90 | 1.78*** | 1.63-1.94 |
| **Recipient BMI (**kg/m^2^) |  | |  | |
| <18.5 | Ref | | Ref | |
| 18.5-24.9 | 1.58*** | 1.38-1.81 | 0.80** | 0.68-0.92 |
| 25-29.9 | 2.12*** | 1.85-2.44 | 0.84* | 0.72-0.99 |
| >30 | 2.40*** | 2.08-2.77 | 0.97 | 0.82-1.14 |
| **Recipient comorbidities** |  | |  | |
| Diabetes mellitus | 2.7*** | 2.61-2.98 | 1.60*** | 1.40-1.82 |
| Coronary artery disease | 2.58*** | 2.40-2.78 | 1.29*** | 1.18-1.40 |
| Peripheral vascular disease | 2.89*** | 2.63-3.17 | 1.42*** | 1.27-1.58 |
| **First KRT modality** |  | |  | |
| Haemodialysis | Ref | | Ref | |
| Peritoneal dialysis | 0.96 | 0.91-1.02 | 1.03 | 0.97-1.10 |
| Pre-emptive | 0.54*** | 0.47-0.61 | 0.73*** | 0.63-0.83 |
| **HLA mismatch** |  |  |  |  |
| 0 Mismatch | Ref | | Ref | |
| 1-3 Mismatch | 1.23*** | 1.08-1.40 | 1.21** | 1.06-1.39 |
| 4-6 Mismatch | 1.36*** | 1.19-1.55 | 1.37*** | 1.19-1.57 |
| **Transplant era** |  | |  | |
| 1989-1998 | Ref | | Ref | |
| 1999-2008 | 0.74*** | 0.70-0.79 | 0.72*** | 0.67-0.77 |
| 2009-2018 | 0.65*** | 0.60-0.711 | 0.47*** | 0.42-0.51 |
| 2018-2021 | 0.38*** | 0.27-0.52 | 0.23*** | 0.16-0.32 |
| **Abbreviations**: ADPKD = autosomal dominant polycystic kidney disease, BMI = body mass index, KRT = kidney replacement therapy, ref = reference, uESKD = kidney failure of unknown aetiology  Significance level: *<0.05, **<0.01, ***<0.001 | | | | |
