# Supplementary material for: Characteristics and clinical outcomes of patients with kidney failure of unknown aetiology from ANZDATA registry
Source: PLoS One. 2024 Mar 11;19(3):e0300259. doi: 10.1371/journal.pone.0300259 (PMC10927112; doi:10.1371/journal.pone.0300259)
Supplement: S6 Table — (DOCX) [file pone.0300259.s006.docx]

**Table S6: Subgroup analysis evaluating association between kidney disease status and kidney transplantation in KRT cohort**

| **Effect** | **Unadjusted** | | **Adjusted** | |
| --- | --- | --- | --- | --- |
|  | **HR** | **95% CI** | **HR** | **95% CI** |
| **Disease status** |  | |  | |
| uESKD | 1.02 | 0.95-1.09 | 1.04 | 0.96-1.2 |
| Diabetic nephropathy | 0.75*** | 0.71-0.79 | 0.81*** | 0.75-0.89 |
| Glomerular disease | 1.03* | 1.0-1.07 | 1.07*** | 1.03-1.11 |
| ADPKD | 1.43*** | 1.36-1.49 | 1.19*** | 1.13-1.25 |
| Other | Ref | | Ref | |
| **Gender** |  | |  | |
| Male | Ref | | Ref | |
| Female | 0.85*** | 0.83-0.88 | 0.84*** | 0.82-1.01 |
| **Ethnicity** |  | |  | |
| White | Ref | | Ref | |
| Non-white | 1.0 | 0.97-1.03 | 0.93*** | 0.90-0.97 |
| **Age** |  | |  | |
| < 20 Years | Ref | | Ref | |
| 20-39 Years | 0.87*** | 0.82-0.92 | 0.95 | 0.89-1.01 |
| 40-59 Years | 1.26*** | 1.19-1.32 | 1.26*** | 1.18-1.35 |
| 60-79 Years | 0.89*** | 0.84-0.95 | 0.83*** | 0.77-0.90 |
| **Smoking status** |  | |  | |
| Never | Ref | | Ref | |
| Former | 1.03 | 0.99-1.06 | 1.01 | 0.98-1.11 |
| Current | 0.83*** | 0.80-0.88 | 0.90*** | 0.55-0.94 |
| **BMI (**kg/m^2^) |  | |  | |
| <18.5 | Ref | | Ref | |
| 18.5-24.9 | 1.04 | 0.98-1.10 | 1.04 | 0.98-1.11 |
| 25-29.9 | 1.27*** | 1.19-1.35 | 1.13*** | 1.05-1.21 |
| >30 | 1.28*** | 1.20-1.36 | 1.04 | 0.97-1.12 |
| **Comorbidities** |  | |  | |
| Diabetes mellitus | 0.76*** | 0.73-0.79 | 0.76*** | 0.71-0.82 |
| Coronary artery disease | 0.68*** | 0.65-0.71 | 0.80*** | 0.76-0.84 |
| Peripheral vascular disease | 0.62*** | 0.58-0.66 | 0.85*** | 0.80-0.91 |
| **First KRT modality** |  | |  | |
| Haemodialysis | Ref | | Ref | |
| Peritoneal dialysis | 1.24*** | 1.20-1.28 | 1.24*** | 1.20-1.28 |
| Pre-emptive | 2.16*** | 2.06-2.25 | 1.53*** | 1.46-1.61 |
| **KRT onset year** |  | |  | |
| 1989-1998 | Ref | | Ref | |
| 1999-2008 | 30.7*** | 2.9-3.2 | 3.6*** | 3.4-3.8 |
| 2009-2018 | 23.8*** | 22.4-25.4 | 32.5*** | 30.4-34.9 |
| 2018-2021 | 304.5*** | 266.7-347.7 | 466.3*** | 404.2-538.0 |
| **Abbreviations**: ADPKD = autosomal dominant polycystic kidney disease, BMI = body mass index, KRT = kidney replacement therapy, ref = reference, uESKD = kidney failure of unknown aetiology  Significance level: *<0.05, **<0.01, ***<0.001 | | | | |
