# Supplementary material for: Characteristics and clinical outcomes of patients with kidney failure of unknown aetiology from ANZDATA registry
Source: PLoS One. 2024 Mar 11;19(3):e0300259. doi: 10.1371/journal.pone.0300259 (PMC10927112; doi:10.1371/journal.pone.0300259)
Supplement: S7 Table — (DOCX) [file pone.0300259.s007.docx]

**Table S7: Death-censored kidney transplantation competing risk analysis**

| **Effect** | **Unadjusted** | | **Adjusted** | |
| --- | --- | --- | --- | --- |
|  | **HR** | **95% CI** | **HR** | **95% CI** |
| **Disease status** |  | |  | |
| uESKD | 1.27*** | 1.22-1.32 | 1.24*** | 1.18-1.29 |
| Known-ESKD | Ref | | Ref | |
| **Gender** |  | |  | |
| Male | Ref | | Ref | |
| Female | 0.99 | 0.97-1.01 | 1.09*** | 1.07-1.12 |
| **Ethnicity** |  | |  | |
| White | Ref | | Ref | |
| Non-white | 0.86*** | 0.84-0.88 | 0.94*** | 0.92-0.96 |
| **Age** |  | |  | |
| < 20 Years | Ref | | Ref | |
| 20-39 Years | 2.24*** | 1.99-2.52 | 2.1*** | 1.84-2.38 |
| 40-59 Years | 5.90*** | 5.26-6.61 | 4.9*** | 4.39-5.62 |
| 60-79 Years | 13.6*** | 12.1-15.2 | 10.6*** | 9.37-12.01 |
| **Smoking status** |  | |  | |
| Never | Ref | | Ref | |
| Former | 1.43*** | 1.40-1.46 | 1.13*** |  |
| Current | 1.36*** | 1.32-1.40 | 1.49*** | 1.45-1.54 |
| **BMI (**kg/m^2^) |  | |  | |
| <18.5 | Ref | | Ref | |
| 18.5-24.9 | 1.26*** | 1.19-1.32 | 0.71*** | 0.67-0.75 |
| 25-29.9 | 1.35*** | 1.29-1.42 | 0.66*** | 0.62-0.70 |
| >30 | 1.39*** | 13.2-1.46 | 0.68*** | 0.64-0.72 |
| **Comorbidities** |  | |  | |
| Diabetes mellitus | 1.85*** | 1.82-1.88 | 1.56*** | 1.53-1.60 |
| Coronary artery disease | 2.56*** | 2.51-2.61 | 1.44*** | 1.41-1.48 |
| Peripheral vascular disease | 2.33*** | 2.29-2.34 | 1.34*** | 1.31-1.37 |
| **First KRT modality** |  | |  | |
| Haemodialysis | Ref | | Ref | |
| Peritoneal dialysis | 0.94*** | 0.93-0.96 | 0.99 | 0.97-1.01 |
| Pre-emptive | 0.13*** | 0.11-0.14 | 0.27*** | 0.24-0.31 |
| **KRT onset year** |  | |  | |
| 1989-1998 | Ref | | Ref | |
| 1999-2008 | 1.03** | 1.00-1.05 | 0.85*** | 0.82-0.87 |
| 2009-2018 | 0.85*** | 0.83-0.88 | 0.66*** | 0.64-0.68 |
| 2018-2021 | 0.50*** | 0.46-0.54 | 0.40*** | 0.37-0.44 |
| **Abbreviations**: ADPKD = autosomal dominant polycystic kidney disease, BMI = body mass index, KRT = kidney replacement therapy, ref = reference, uESKD = kidney failure of unknown aetiology  Significance level: *<0.05, **<0.01, ***<0.001 | | | | |
