# Supplementary material for: Characteristics and clinical outcomes of patients with kidney failure of unknown aetiology from ANZDATA registry
Source: PLoS One. 2024 Mar 11;19(3):e0300259. doi: 10.1371/journal.pone.0300259 (PMC10927112; doi:10.1371/journal.pone.0300259)
Supplement: Attachment — Submitted filename: uESKD KF_Response To Reviewers_PLOSOne Resubmission2.pdf [file pone.0300259.s010.pdf]

Responses to Editorial and Reviewer Queries PONE-D-23-34616R1
"Characteristics and clinical outcomes of patients with kidney failure of unknown aetiology from ANZDATA registry"

Editorial Team

1. Thank you for response to our reviewers' comments. Still, some minor adjustments need to be made before final acceptance. Please address the reviewers' comments and send the manuscript back.

Thank you so much for your and the Reviewers' time and input which we greatly value. We have addressed the additional queries below.

Reviewer #1

2. Thank you for the responses and congratulations to the authors. Most comments have been well addressed. I have no further comments for the authors.

We appreciate your input and previous queries.

Reviewer #2

3. You classified them as uESKD and non-uESKD. In my opinion, it would sound better to replace non-uESKD with known ESKD.

This is a good point which we take on board. The first instance of mentioning this in the abstract and main manuscript we have used the term "*non-uESKD (known-ESKD)*" to signify what this group is and then used "*known-ESKD*" thereafter.

4. In the results, you stated that the median follow-up time for the dialysis was 3 years. This indicates that at least 50% of the patients were followed for only 3 years. Then you mentioned that the mortality rate for people on dialysis at 5 years was 53.1%. I think these numbers need to be revised.

Thank you for astutely pointing this out. We have investigated further and recalculated the median follow-up times using the reverse Kaplan-Meier estimator and have updates the results and statistical methodology accordingly. This methodology is more appropriate for calculating median follow-up times in cohorts such as this, and we thank the reviewer for identifying this. (New methods for estimating follow-up rates in cohort studies - PMC (nih.gov, PharmaSUG-2019-ST-081.pdf)

5. In conclusion; uESKD has similar likelihood of kidney transplantation compared to uESKD. Please revise and correct this sentence.

We have corrected this.
